# What makes Sanriku waters the southernmost habitat of northern fur seals? Winter–spring habitat use in relation to oceanographic environments

**DOI:** 10.1371/journal.pone.0287010

**Published:** 2023-06-21

**Authors:** Yu Kanaji, Hiroto Murase, Shiroh Yonezaki

**Affiliations:** 1 Fisheries Resources Institute, Japan Fisheries Research and Education Agency, Yokohama, Kanagawa, Japan; 2 The Institute of Cetacean Research, Tokyo, Japan; 3 The Tokyo University of Marine Science and Technology, Tokyo, Japan; Hawaii Pacific University, UNITED STATES

## Abstract

The waters off Sanriku (located on the northeastern coast of Honshu Island, Japan) provide the southernmost habitats of northern fur seals (*Callorhinus ursinus*) during winter and spring in the western North Pacific. The southward flowing cold Oyashio current and northward-flowing warm Kuroshio extension mix there, making the area highly productive. Northern fur seals migrate into these waters from the breeding rookeries for feeding, and the locations of the southern margins of their habitats vary yearly. The key questions for understanding the seasonal migration patterns are “why” and “how” the species utilize these waters as the southernmost habitat. We estimated the density and abundance of northern fur seals using standard line-transect theory combined with habitat modeling. The spatial patterns of animal density were analyzed using generalized additive models with seven static and dynamic environmental covariates, and those covariates were selected based on Akaike’s information criterion (AIC). The lowest AIC model included depth, sea surface temperature, slope, and gradient in sea surface temperature. This model estimated well the spatial patterns of the density of the species, in which fur seals were widely distributed in the study areas, but less frequently encountered between the isobaths 100 m and 200 m. These spatially separated habitats suggest that the shelf break and offshore front play an important role in creating the feeding grounds of fur seals. On the other hand, sea surface temperature positively correlated with fur seals’ density up to 14°C. This may indicate that further warm waters work as a temperature barrier, and fur seals concentrate on the edge of suitable temperature ranges.

## Introduction

The waters off Sanriku (located on the northeastern coast of Honshu Island, Japan) are known to be one of the most productive fishing grounds in the western North Pacific. The southward-flowing cold Oyashio current and northward-flowing Kuroshio extension mix there, transporting both subarctic species (e.g., chum salmon *Oncorhynchus keta* and walleye pollock *Theragra chalcogramma*) and species migrating from temperate waters (e.g., Japanese anchovy *Engraulis japonicus*, chub mackerel *Scomber japonicus*, and Japanese common squid *Todarodes pacificus*), and provide key habitats for these pelagic species [1,2]. This so-called Kuroshio-Oyashio mixing water is oceanographically dynamic, where bifurcated energetic jets and mesoscale eddies supply nutrients to sustain high phytoplankton production [3–6]. Many oceanic top predators, such as marine mammals, also utilize these productive waters. The northern fur seal *Callorhinus ursinus* is one of the most abundant mammalian species in Sanriku during winter and spring [7].

Previous analyses involving design-based line-transect modeling estimated several thousand northern fur seals migrating into this area annually, while the estimated abundances were highly variable year-by-year [7]. In a conventional design-based approach, animal density along predetermined track lines is extrapolated to the entire survey area [8], while it is biologically plausible that animal density is dependent on ambient environments. The factors relating to such variability are key to understanding the mechanism by which fur seals select their habitat in this region. Northern fur seals migrate from breeding rookeries in Tuleny, Commander, and Pribilof Islands in northern subarctic waters to Sanriku during winter [9]. Wada [10] analyzed the records of northern fur seal observations and reported that fur seals migrate south during winter, then arrive off the Sanriku coast in late February and stay through March and April [10]. The Sanriku waters comprise their southernmost range, while the locations of the southern limits of their habitats vary from year-to-year at around 36–38°N, corresponding to the position of the oceanic front between the warm Kuroshio and cold Oyashio currents [10,11]. Fur seals started northward migration in late spring, and almost all animals moved out from that region in late June, although they are still widely distributed on the southern coast of Hokkaido in late May [10]. Adult females aged four years or older mainly inhabited the southernmost area of the distribution range, while adult males aged eight years or younger migrated to northern Sanriku, north of 38°N [10,11]. Fur seals exhibit opportunistic feeding, so the distribution and abundance of all available prey species rather than any specific preferred prey species indicate the habitat suitability of fur seals [2,12]. Japanese sardine (*Sardinops melanostictus*), chub mackerel (*S*. *japonicus*), and myctophid fishes have been recognized to be dominant prey species in the Sanriku waters [2,13,14]. The habitat ranges of these prey species are known to relate to topography [14,15]. For example, Japanese sardine was more frequently predated by fur seals in shelf waters, while Japanese common squid and sparkling enope squids were more frequently predated in offshore and slope waters, respectively [14]. Those studies generally depended on the visual inspection on the maps simply overlaying sighting positions of fur seals and prey and temperature distributions. Such conventional approaches have hardly provided direct information on animal density, abundance, and distributional patterns in their habitats. Nowadays, habitat modeling techniques enable us to analyze quantitively and objectively the relationship between species distribution and environmental factors [16–18].

The objective of the present study was to estimate spatial distribution patterns of northern fur seals in the southernmost habitat in Sanriku during winter and spring in relation to oceanographic environments. A standard analytical approach composed of generalized additive and line-transect models was applied to the dataset from sighting surveys [16]. Such a habitat modeling approach also provides abundance estimates in the survey area. We selected environmental covariates that significantly affect their distribution and created spatial distribution maps showing animal density using these variables. Based on the results, we tested the hypotheses that distributional patterns in their southernmost habitat are related to topography and seawater temperature.

## Materials and methods

### Survey data and environmental variables

**Fig 1:**
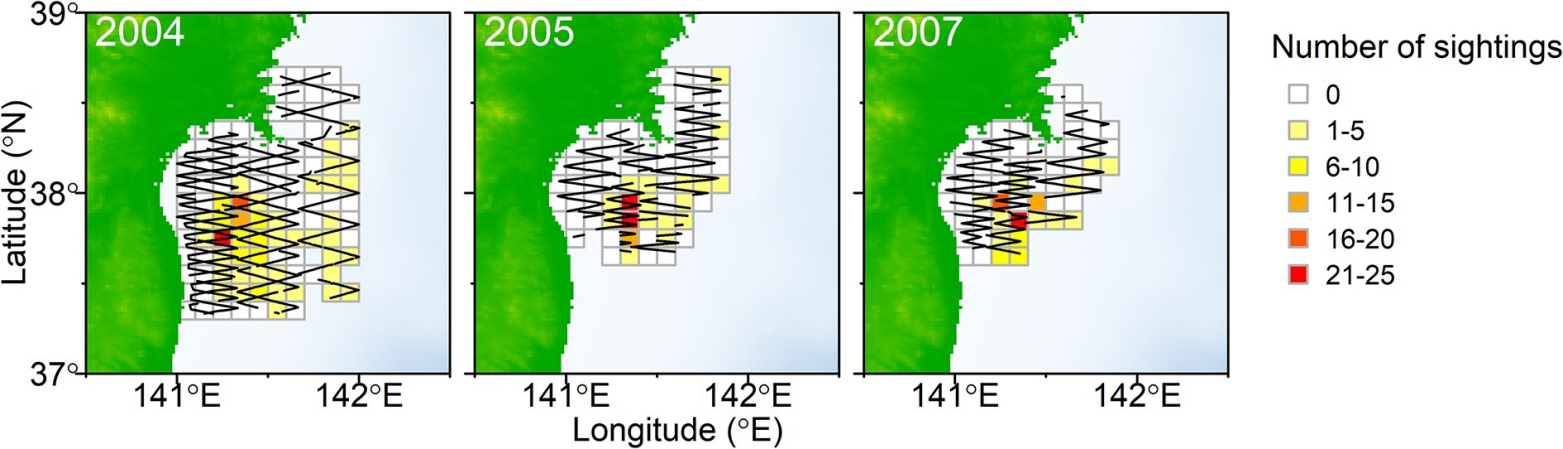
The grid cells of 0.1° longitude × 0.1° latitude showing the number of northern fur seal sightings. Lines represent survey track lines.

All sightings data used were previously published by Kanaji et al. [7], who estimated the abundance of northern fur seals using design-based line-transect analysis. When suitable sighting conditions were met (Beaufort scale ≤4), a total of 1064.1, 558.4, and 538.9 nautical miles (nmi ≈ 1970.7, 1034.2, and 998.0 km) were surveyed in 2004, 2005, and 2007, respectively. The surveys were conducted from April 12 to May 11 in 2004, April 11–28th in 2005, and April 9–27 in 2007; during these surveys, a total of 186, 83, and 219 fur seal groups were recorded during on-effort surveys, respectively. The survey areas differed by year (Fig 1). Those in 2005 and 2007 were smaller than that in 2004 because the former two surveys were ecosystem surveys that included time-consuming net sampling, while the survey in 2004 was a dedicated sighting survey. For further details on survey protocol, see Kanaji et al. [7].

**Fig 2:**
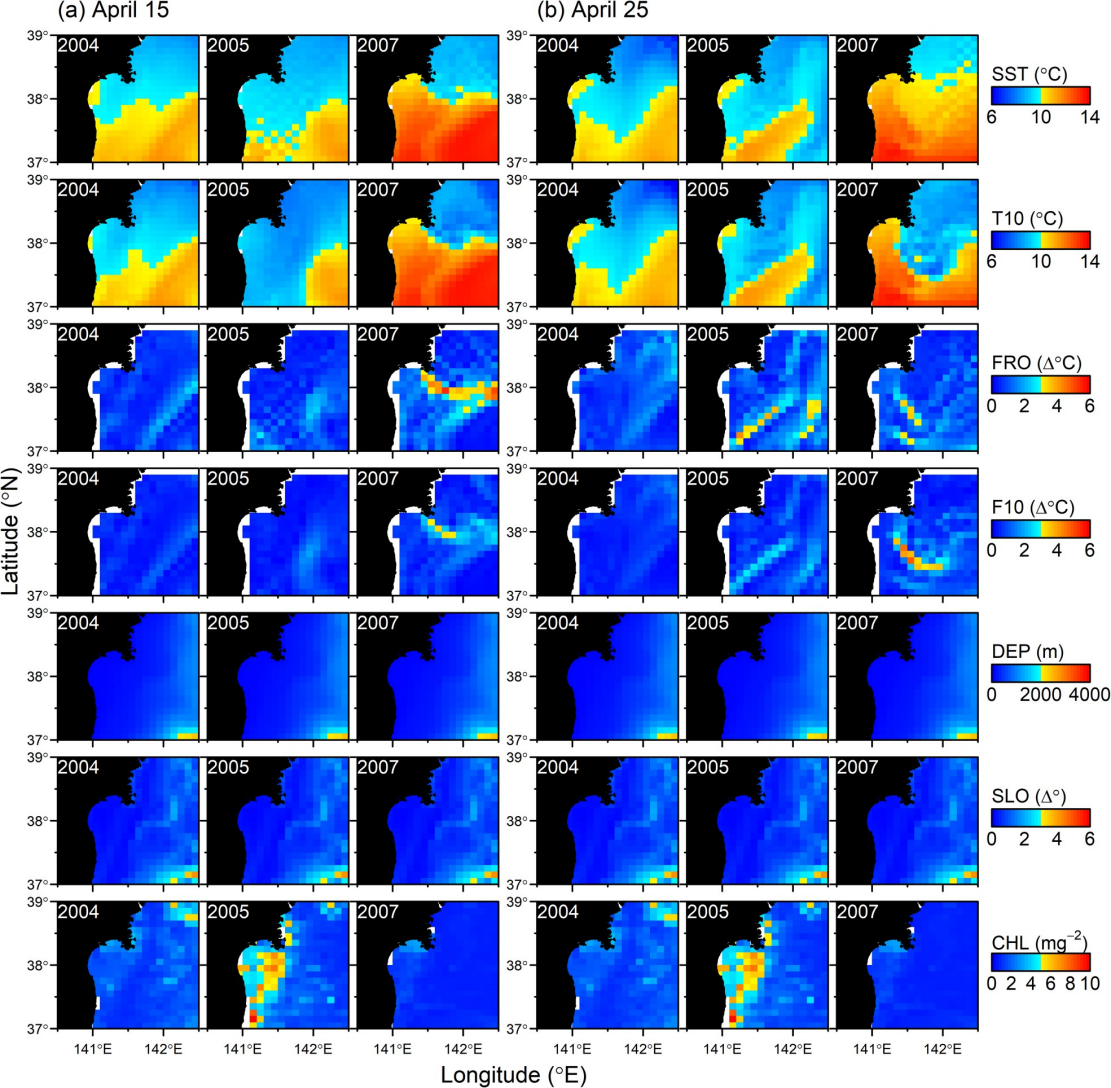
Distributions of seven environmental variables, sea surface temperature (SST), temperature at 10m deep (T10), gradient in SST (FRO) and T10 (F10) as an indicator of oceanic fronts, bottom depth (DEP), gradient in DEP as an indicator of slope (SLO), and chlorophyll a concentration (CHL).

The study areas were divided into grid cells of 0.1° longitude × 0.1° latitude (Fig 1). The number of group sightings and effort length were divided into the cells. The environmental variables were associated with them. Because shifts in isotherms and topography-related feeding habits have been known to affect seal’s seasonal distribution [10,11,14], the following seven environmental variables were investigated (Fig 2): sea surface temperature (SST), the temperature at 10m deep (T10), the gradients in SST (FRO) and T10 (F10) as an indicator of oceanic fronts, bottom depth (DEP), the gradient in DEP as an indicator of slope (SLO), and chlorophyll a concentration (CHL). Daily SST and T10 data were obtained from the Japan Fisheries Research and Education Agency Regional Ocean Modeling System II (FRA-ROMS II), which contains environmental variables at a spatial resolution of 0.1° longitude × 0.1° latitude and a temporal resolution of 1 day [19]. Longitudinal and latitudinal differences in temperature values in neighboring grids were used to calculate FRO and F10 as ${\left({\Delta SST}_{lon}^{2}+{\Delta SST}_{lat}^{2}\right)}^{0.5}$. DEP data was obtained from ETOPO1, a 1-arc-minute global relief model [20], and SLO was calculated using DEP differences in degrees between neighboring grids in longitudinal and latitudinal directions as ${\left({\Delta DEP}_{lon}^{2}+{\Delta DEP}_{lat}^{2}\right)}^{0.5}$. Then those values were compiled into 0.1° resolution by averaging within each grid. Monthly CHL data was obtained from ocean color images (https://oceancolor.gsfc.nasa.gov/; accessed April 1, 2021). We used the data set of monthly averaged CHL because the ocean color images often include missing data caused by clouds; but the missing data area was filled out using the monthly data set. As well as these environmental variables, we tested the effect of year.

### Generalized additive models (GAMs) and abundance estimation

The basic structure of the generalized additive models (GAMs) to estimate the encounter rate (group sightings per unit effort) was:

$${\hat{\lambda }}_{i}=exp\left[ln(l_{i})+x_{0}+\sum_{k}f_{k}(z_{ik})\right]$$ (1)

where ${\hat{\lambda }}_{i}$ is the expected number of the group sightings within the *i*-th grid, ln(*l*_*i*_) is the offset term of the effort length, *x*_0_ is the intercept, *f*_*k*_ is the spline function for the *k*-th explanatory variable *z*_*ik*_; SST, T10, FRO, F10, DEP, SLO, and CHL. GAM-based habitat models are likely to be sensitive to a few extreme outliers [18]. Before the analyses, we checked for outliers by testing the above linear model using the glm function in R [21] and visually inspected Cook’s distance. Only one data point was truncated by the 99.9th percentile applied to the encounter rate. The negative binomial distribution was used to express error structures in this model.


$$n_{i}∼Negative\;binomial({\hat{\lambda }}_{i},\phi )$$ (2)

Negative binomial distribution with dispersion parameter *ϕ* is commonly used for habitat modeling of the data from cetacean sighting surveys because the encounter rate is likely to be over-dispersed [18,22]. The function gam in R package mgcv v.1.8-35 was used to estimate the model [23]. Variable selection was conducted using a forward stepwise approach based on Akaike’s information criterion (AIC). The models with the same variables at different depth layers (e.g., SST and T10) were not considered candidate models in this process because those values were highly correlated (see Results).

Abundance within *i*-th grid cell ${\hat{p}}_{i}$ was estimated by:

$${\hat{p}}_{i}=\frac{a_{i}{\hat{\lambda }}_{i}\bar{s}}{{2l}_{i}{\hat{w}}_{i}}$$ (3)

where *a*_*i*_ is the surface area, $\bar{s}$ is the mean group size, and ${\hat{\lambda }}_{i}/l_{i}$ is the encounter rate estimated from Eqs 1 and 2. We simply used yearly mean group sizes for $\bar{s}$ in this study. It is also possible to estimate group size in relation to environmental covariates. However, in the distance sampling approach, sampling of group size in not random, because small size group tends to be missed when group is detected far from the track line [8]. To remove such bias depending on detection distance, detection probability (*g*(*x*)) (or detection perpendicular distance *x*) must be included in a linear model together with environmental covariates (e.g. [18]). This is often difficult because the relationship between group size and *g*(*x*) is usually already modeled by the detection function (see below).

The effective strip half-width (esw, ${\hat{w}}_{i}$) was estimated by integrating the detection function that describes the detection probability at a given perpendicular distance [8]. Here, we considered two types of detection functions:

$$Half\;normal:\;g(x_{j})=\exp\left(-\frac{x_{j}^{2}}{2exp{\left(\alpha_{0}+\sum_{m}\alpha_{m}\gamma_{jm}\right)}^{2}}\right)$$ (4)

$$Hazard\;rate:\;g(x_{j})=1-\exp\left(-{\left(\frac{x_{j}}{\exp\left(\alpha_{0}+\sum_{m}\alpha_{m}\gamma_{jm}\right)}\right)}^{-\theta }\right)$$ (5)

Here, *x*_*j*_ is a perpendicular distance for the *j*-th sighting. The parameter *α*_0_ is the intercept, and *α*_*m*_ is the coefficient associated with *m*-th covariate *γ*_*jm*_ potentially affecting detection probability [24]. Three covariates, namely year, observed group size, and Beaufort scale, were considered in the multivariable detection function, and the models with combinations of these covariates were compared based on the AIC. When these detection functions are divided by their integrals, detection functions themselves work as probability density function [8]. As such, these parameters can be estimated in the framework of the maximum likelihood approach. These detection functions were estimated using function ds in the R package Distance v.1.0.5 [16].

Habitat prediction maps were created by projecting the estimated models to the environmental variables on April 15 and 25 each year because these dates were covered by all three years. The total abundance *P* of northern fur seals migrating into Sanriku waters was calculated by summing ${\hat{p}}_{i}$ across the entire survey area. To evaluate the uncertainty of *P*, a nonparametric bootstrap method was applied. We randomly selected grid cells with replacement (i.e., each grid cell can be selected multiple times) and then estimated the GAM and the detection function for calculating the total abundance. For both the GAM and detection function, the covariates selected above were directly used (i.e., the AIC-based model selection process was not applied here). We finally obtained 1000 sets of bootstrapped samples, which were used to estimate the uncertainty of the total abundance.

## Results

After segmenting sighting survey data into grid cells of 0.1° longitude × 0.1° latitude, the numbers of sightings in each grid are summarized in Fig 1. There were relatively fewer sightings in the areas north of 38°N and shallow coastal areas then in the southwestern regions. Segmented sighting and effort data was associated with environmental variables daily. Therefore, when a certain cell at a given location was surveyed by multiple days, it generated multiple daily data from the same location cell. After removing one outlier and grid cell with missing environmental variables (e.g., shallow coastal areas), we finally obtained 341 grid cells with 370 group sightings and 1954.6 nmi (≈3619.8 km) on-effort track lines (Table 1).

**Table 1:** Summary of the dataset used for GAM analyses.

Year	Date	Effort (nmi)	Effort (km)	No. of group sightings	No. animal sightings	No. of grids with one or more sightings	No. of grids wuithout any sightings
2004	April 12–May 11	976.2	1807.9	186	1146	61	128
2005	April 11–28	520.3	963.6	83	222	20	65
2007	April 9–27	458.1	848.3	101	391	17	50

**Fig 3:**
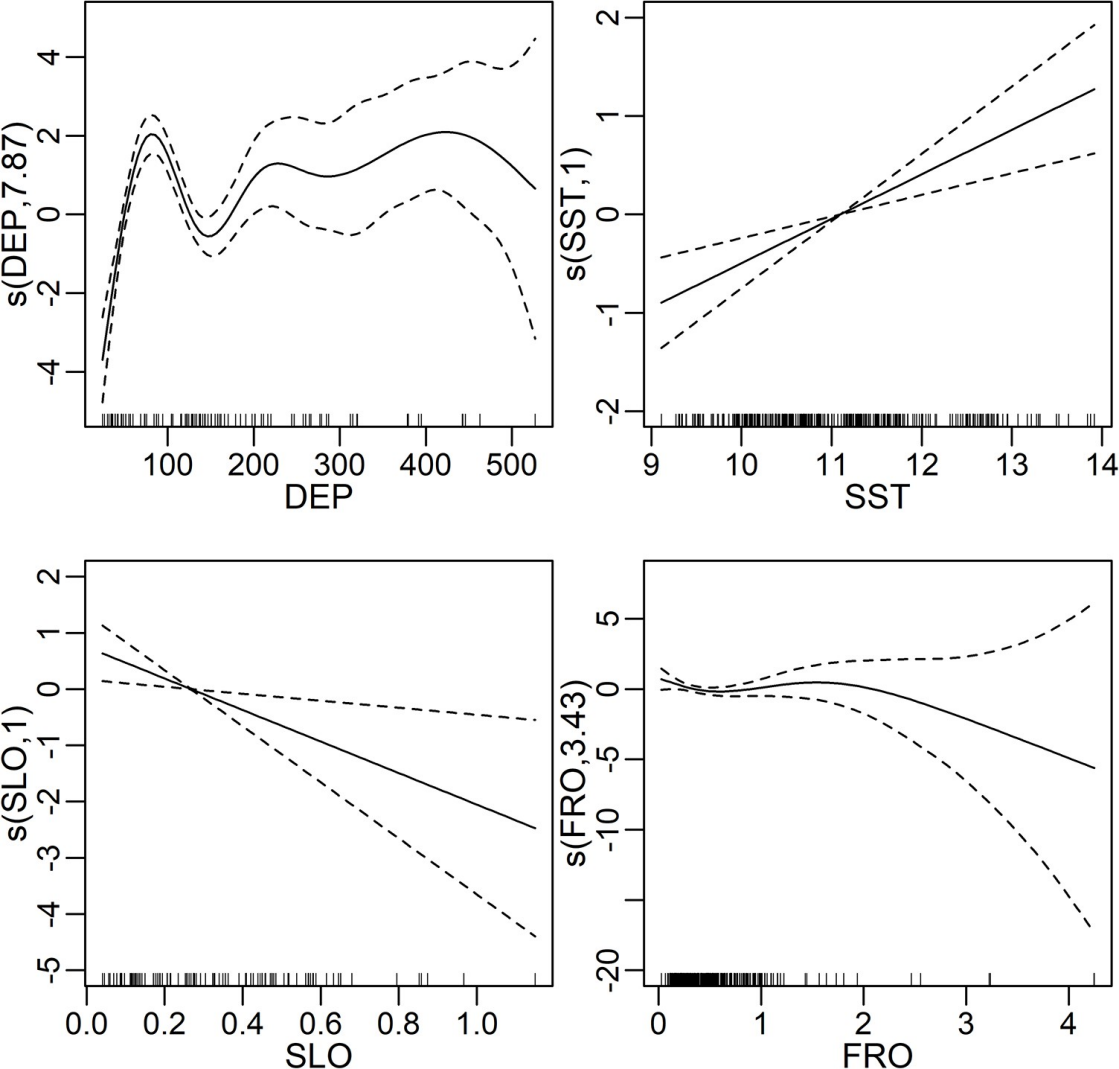
Functional response curves of the generalized additive model (GAM) with the four environmental variables selected as the lowest AIC model: Bottom depth (DEP), sea surface temperature (SST), gradient in DEP as an indicator of slope (SLO), and gradient in SST (FRO).

We constructed GAMs using combinations of environmental variables. The correlation among seven variables is summarized in S1 Fig. A Pearson’s correlation coefficient > 0.7 is considered a high correlation. According to this general criterion, we did not consider the models that included both SST and T10 (correlation coefficient = 0.83) or those that included both FRO and F10 (0.81) for the model selection. When single variable was modeled, the model with lowest AIC had a variable of DEP (AIC = 743.5) (S2 Fig). As a result of stepwise forward selection, the GAM with the variables of DEP, SST, SLO, and FRO with the lowest AIC model was selected. The AIC of this model was improved to 717.9. Functional response curves are shown in Fig 3. The encounter rate for northern fur seals tended to be larger in higher SST and smaller SLO values. Two modes of encounter rate were detected at around DEPs of 100m and 200–400m (Fig 3). There were also two modes at around 0 and 2 of SLO values (Fig 3).

**Fig 4:**
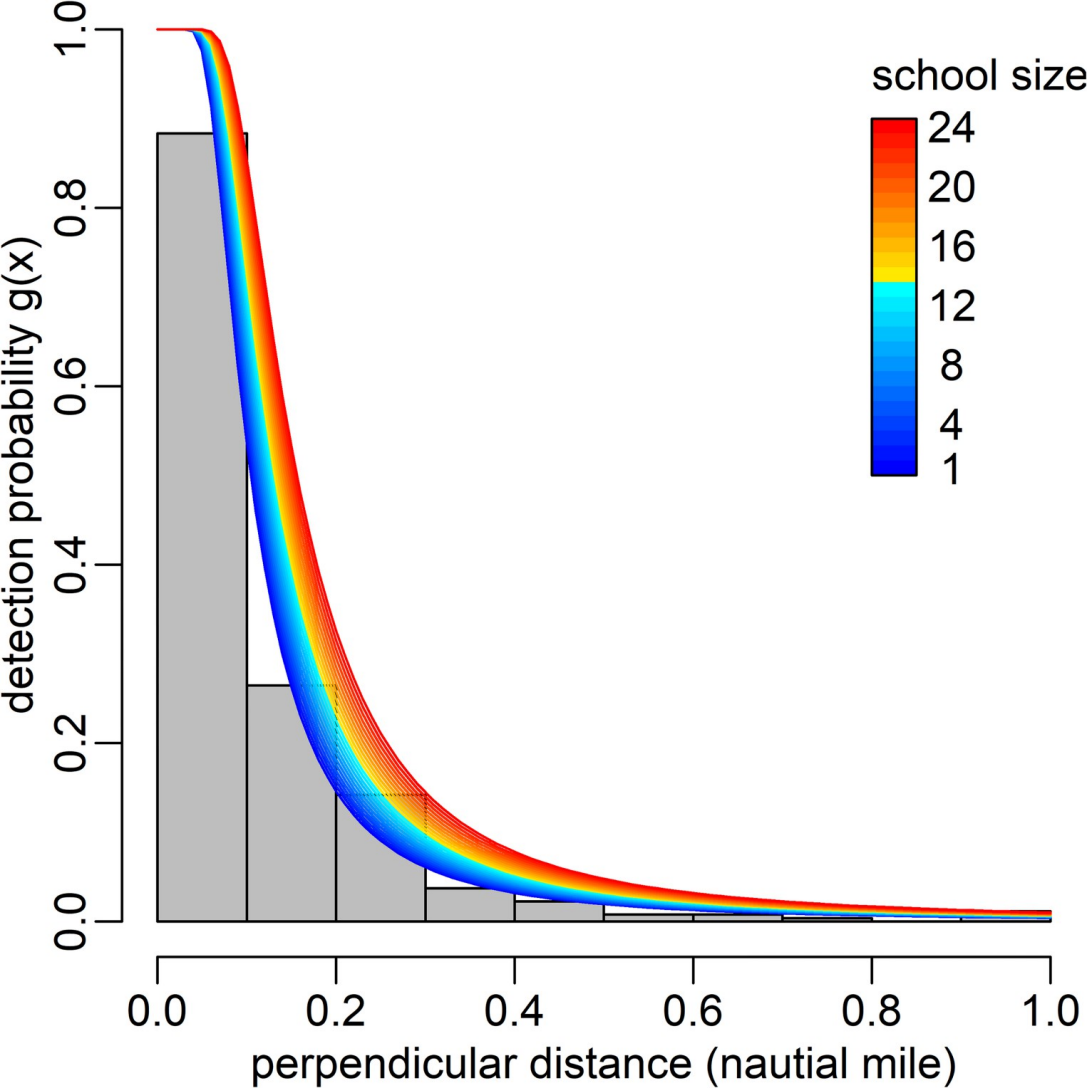
Histogram of the perpendicular distances of northern fur seal sightings and hazard-rate detection function with group size covariate.

Half-normal and hazard-rate models with multiple covariates were fitted to the perpendicular distances to the fur seal groups (Fig 4). The AIC of the hazard-rate model with group size covariate was −838.4, being the lowest value among the candidate models (e.g., −706.8 for half-normal and −838.2 for hazard-rate models without any covariates). The esw ($\hat{w}$) estimated by this lowest AIC model was 0.14, 0.15, and 0.19 nmi (≈2.26, 0.27, and 0.35 km) when group size = 1, 5, and 20, respectively (Fig 4). Mean group sizes ($\bar{s}$) were 6.2, 2.7 and 3.9 in 2004, 2005 and 2007, respectively.

**Fig 5:**
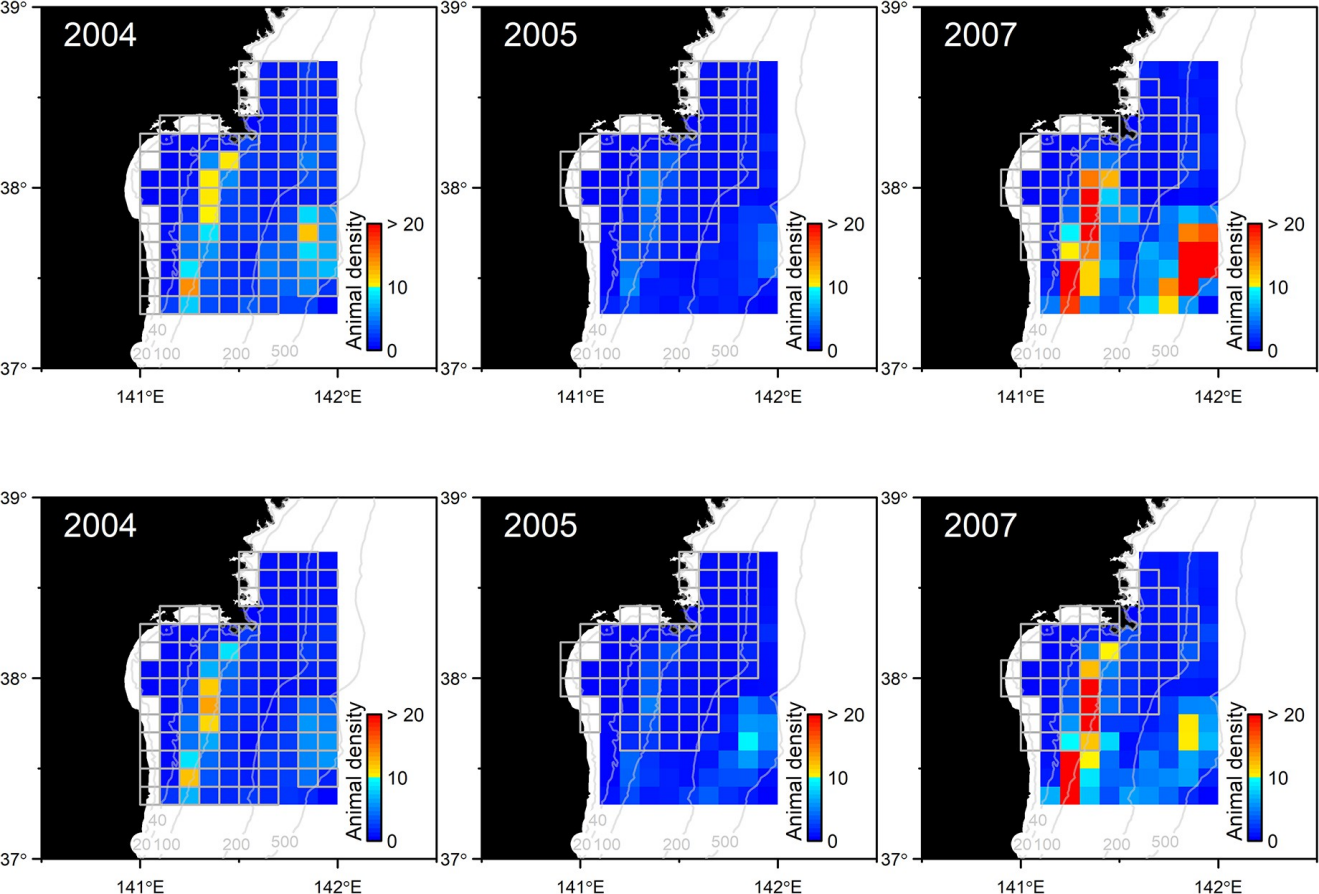
Habitat prediction maps based on the generalized additive models projected to the surfaces of four environmental variables (DEP, SST, SLO, and FRO) on April 15 (top) and 25 (bottom) in each year. Contours represent isobaths of 20, 40, 100, 200, and 500 m.

The GAM constructed was projected to the set of environmental variables on April 15 and 25 in 2004, 2005, and 2007, and then animal density ($\hat{p}/a$) was spatially estimated using the estimated esw and mean group size (Fig 5). In the spatial distribution maps, two main habitats were detected in each year. One was a coastal habit along 100m isobath, and another was an offshore habitat ranging between 200–500m isobaths (Fig 5). Both could be seen in all three years, but those densities and distribution varied over the years. In coastal and offshore habitats, animal density was higher in 2007 than in other years. In 2007, areas with high animal density tended to be in slightly south of the other years.

Abundance (${\hat{p}}_{i}$) was calculated by multiplying the density and area in each grid cell. Then, the total abundances (coefficients of variation) within the area surveyed were estimated to be 8559 (0.49), 1802 (0.37) and 5068 (0.48) on April 15 in 2004, 2005, and 2007, respectively, whereas those for April 25 were 6731 (0.50), 1450 (0.37), and 4594 (0.40), respectively.

## Discussion

Our results effectively captured the high-density areas of northern fur seals (Fig 5). In particular, fur seals were widely distributed in the study areas, but less frequently encountered between 100m and 200m isobaths (Fig 5). The four variables were selected by AIC, and AIC was increased from 717.9 to 720.8 when FRO was dropped from the final model. Nevertheless, FRO showed relatively weaker correlation with group density when it was modeled as a single variable (S2 Fig). Pair-wise correlation of FRO with any other variables tended to be weak (S1 Fig), so the variable was possibly well explaining unexplained variances by the other three variables. In fact, relatively higher FRO values were observed in two separate areas particularly in 2004, which well coincided with high density areas of northern fur seals (Fig 1). These spatially separated habitats were also estimated well by DEP (Fig 3). Kiyota and Yonezaki [14] pointed out that the feeding habits of northern fur seals differed between sampling locations on the continental shelf (depth of ≤200 m), slope (201–1000 m), and offshore (> 1000 m) areas. Therefore, the distributional patterns of northern fur seals are likely related to the distribution and abundance of prey species. It is difficult to estimate the relationship between prey and fur seal distributions directly because they feed opportunistically on a wide variety of prey species, mostly at night and dawn [2,14], and the sighting surveys in this study were conducted during in the daytime. However, the coastal habitat overlapped widely with high-density areas of Japanese anchovy in 2005 [15]. Kiyota and Yonezaki [14] also pointed out that the Japanese anchovy was an important prey species for northern fur seals inhabiting shelf waters. This fact does not necessarily imply that the density of anchovy is a good predictor of the distribution of northern fur seals. The estimates by Murase et al. [15] still have uncertainty because they were obtained from the data sets only in a year and at the beginning of the migration season of Japanese anchovy to the Sanriku waters. In addition, environmental conditions in 2005 were quite different from those of other years; the seawater temperature was relatively colder and the CHL was significantly higher in the areas around the coastal side of the study area (Fig 2). In the GAM constructed in this study, the encounter rate of fur seal groups was positively correlated with SST. No significant relationship was detected with CHL. Abundance was thereby estimated to be smaller in 2005 than in other years.

It might sound contradictory if high productivity and prey availability cause the low density of fur seals. However, our study area was the southernmost habitat, and northern fur seals seasonally migrate from cold subarctic environments. Therefore, it is plausible that high seawater temperature is a predictor of the habitat and works as a barrier for northern fur seals moving further south. In 2005, colder waters extended further south, which could have provided a much-extended habitat southward and relatively sparce density in the study region. On the other hand, in 2007, colder waters were limited to the northern region, which could have caused a concentration of fur seals in specific areas in the study region. The fact that the encounter rate was correlated with a weak front further supports this hypothesis (Fig 3). In this regard, one contradiction was that our GAM showed a positive linear correlation with SST. This is possible because the study area only covered SST up to 14°C, but further warm water might have an adverse effect on the fur seal’s habitat. In fact, Wada [10] pointed out that a SST just below 15°C is a suitable habitat for fur seals, and fur seals groups were scarcely distributed in the waters with 15°C and warmer SST. In addition, estimated abundances tended to be larger on 15 April than on 25 April, which might indicate beginning of northward migration in this season. However, this is not conclusive because spatial shift in environmental variables were not so clear in such a short interval (10 days), and uncertainty (coefficient of variation) for the abundance estimates were also large.

The offshore habitat of northern fur seals in DEPs of 200–400m roughly overlapped with the area where the krill density was higher [15]. This might be related to offshore prey species, such as myctophid fishes. In addition, fur seals’ offshore habitat corresponded well with a weak front extending from southwest to northeast (Fig 2). This frontal structure occurred in all three years.

Kanaji et al. [7] provided abundance estimates of northern fur seals within the same study areas using a conventional design-based approach. Those were 8191, 1288, and 5672 in 2004, 2005, and 2007, respectively, which is a very close to the abundance estimates by this habitat modeling approach (e.g., 8559, 1802, and 5068 April 15). This suggests that year-to-year variations in the abundance of fur seals migrating into the Sanriku waters were well explained by environmental covariates. However, environmental variables were not considered into mean group size estimation, so the cause of those variations remains unclear. Besides, the offshore habitat in DEPs of 200–400m was only covered by the survey in 2004. Our GAM predicted a similar pattern for the other years, but the data could not prove this. Thus, further investigation is needed to cover a wider study area to test whether dynamic oceanography determines the distribution pattern of fur seals. Even with such limitations, our results suggest that the shelf break and offshore front play an important role in the habitat for fur seals. In addition, water temperature controls density in the study area and distribution patterns in much wider spatial scales. These findings and interpretations are consistent with the previous hypothesis, but based on standard habitat modeling approaches and environmental variables, we have now supported it in more objective and reliable ways.

## Conclusions

We estimated the abundance and animal density of northern fur seals and their spatial patterns using line-transect theory and GAM-based habitat models. Our models indicated that the main habitat of northern fur seals was separated into the shelf edge region and the offshore frontal region. Those areas were considered to be related to the availability of the prey species. In addition, the models indicated that animal density increased as SST up to 14°C. Wada [10] previously analyzed the relationship between the sighting locations of fur seals and observed SST, and pointed out that SST just below 15°C is a suitable habitat for fur seals, and that fur seals were scarcely distributed in the waters with 15°C and warmer SST. The total abundance estimated using these habitat-based models was nearly the same as that of conventional line-transect analyses [7], which suggests that variations in oceanographic environments well-explained year-to-year variations in abundance estimates. Northern fur seals migrate into Sanriku waters from breeding rookeries in the subarctic waters during the winter and spring seasons. Therefore, the southern margin of their habitat has been known to change yearly according to changes in oceanographic environments, such as the locations of temperature fronts. Therefore, the formation of the southernmost habitats of northern fur seals appears to be related to physical and biological oceanography, particularly with topography, weak offshore fronts, and temperature barriers in the waters off Sanriku.

## Supporting information

S1 Fig Correlation plots. (DOCX)

S2 Fig Functional response curve. (DOCX)

S1 File Grid data. (CSV)

S2 File Distance data. (CSV)
