# Supplementary material for: What makes Sanriku waters the southernmost habitat of northern fur seals? Winter–spring habitat use in relation to oceanographic environments
Source: PLoS One. 2023 Jun 21;18(6):e0287010. doi: 10.1371/journal.pone.0287010 (PMC10284389; doi:10.1371/journal.pone.0287010)
Supplement: S1 Fig — (DOCX) [file pone.0287010.s001.docx]

S1 Fig. Correlation plots among seven environmental variables, sea surface temperature (SST), temperature at 10m deep (T10), gradient in SST (FRO) and T10 (F10) as indicators of oceanic fronts, bottom depth (DEP), gradient in DEP as an indicator of slope (SLO), and chlorophyll a concentration (CHL) (lower triangular) and Pearson’s correlation coefficients among them (upper triangular).
