# Supplementary material for: What makes Sanriku waters the southernmost habitat of northern fur seals? Winter–spring habitat use in relation to oceanographic environments
Source: PLoS One. 2023 Jun 21;18(6):e0287010. doi: 10.1371/journal.pone.0287010 (PMC10284389; doi:10.1371/journal.pone.0287010)
Supplement: S2 Fig — (DOCX) [file pone.0287010.s002.docx]

S2 Fig. Functional response curves of the eight generalized additive models (GAM) with each single variable (sea surface temperature, SST; temperature at 10m deep, T10; gradients in SST, FRO; gradients in T10, F10; bottom depth, DEP; gradient in DEP, SLO; chlorophyll a concentration, CHL; and a factor of the year). AIC was shown on the top-left in each panel.
